# Psychometric properties of the Bern illegitimate tasks scale using classical test and item response theories

**DOI:** 10.1038/s41598-023-34006-0

**Published:** 2023-05-03

**Authors:** Beata Aleksandra Basinska, Anna Maria Dåderman

**Affiliations:** 1 Faculty of Management and Economics, Gdansk University of Technology, Narutowicza 11/12, 80-233 Gdańsk, Poland; 2 Department of Social and Behavioral Studies, University West, Trollhättan, Sweden

**Keywords:** Psychology, Health occupations

## Abstract

Combining a classical test theory and an item response theory (IRT), this study aimed to investigate the psychometric properties of the Bern Illegitimate Tasks Scale (BITS) by measuring two conceptually separate dimensions capturing unnecessary tasks (perceived by employees as pointless) and unreasonable tasks (perceived as unfairly or inappropriately assigned). Data collected among Polish employees in two samples (*N* = 965 and *N* = 803) were analysed. Results from the classical test theory (parallel analysis, exploratory and confirmatory factor analyses) indicated two correlated factors with four items each, confirming the theory of illegitimate tasks. This study is the first to report item and scale functioning using IRT analysis of each of the two dimensions of BITS. All items on each dimension had acceptable discrimination and difficulty parameters. Moreover, items had measurement invariance between men and women. All levels of unnecessary and unreasonable tasks were reliably captured by BITS items. Convergent and discriminant validities of both dimensions of BITS were confirmed in relation to work overload, work performance and occupational wellbeing. We conclude that BITS, in the case of the Polish version, is psychometrically suitable to use with the working population.

## Introduction

Contemporary organisations operate in a competitive, uncertain and volatile environment^1^. Employees working in such organisations are often asked to perform duties (illegitimate tasks) that are not in agreement with their professional identity^2,3^. They usually report that tasks are illegitimate due to either employees feeling that a task should not have to be carried out by anybody, or that a task could have been avoided with better organizational systems^4^ (unnecessary tasks); or tasks being unfairly assigned to them, that is, “tasks that may be outside the range of an employee’s occupation or status, or professional role”^4^ (p. 73) (unreasonable tasks). In the subjective view of employees (that reflect normative prescriptions of what is expected, or seen as legitimate, in a given role or position), unnecessary tasks are perceived as pointless, whereas unreasonable ones are seen as inappropriate, given employees’ competencies or professional resources. Illegitimate tasks lead to greater levels of administrative work and reduce the time spent on core responsibilities. For instance, rather than focusing on providing medical care to patients, teaching students, producing more research or facilitating citizens’ daily lives, employees may be asked to prepare double documentation (i.e., both on paper and in electronic form), numerous reports, and summaries^5,6^. Illegitimate tasks have been recognised as an important social stressor that threatens the self and violates one’s professional identity, according to Stress-as-Offence-to-Self (SOS) theory^7,8^. SOS theory assumes that maintaining a positive self-view is a primary human concern, and any threat to self-esteem elicits strain^7^. Illegitimate tasks cause stress because employees feel they are not valued and respected when they receive these tasks^3,8^. Thus, recipients feel that either no one should be assigned such tasks because they are pointless or they are left feeling “I should not have to do this! It is not my job!”. Illegitimate tasks can also be understood as job demands due to their energy-related features because they require effort and drain employees’ physical and psychological wellbeing^9^. Therefore, as illegitimate tasks mount over time, employees may lose their professional identity and engagement, which can cause further negative consequences for individuals and organisations^2,10–12^. What is needed, then, is a valid and effective tool that will respond to challenges which appear at rapid speed. The Bern Illegitimate Tasks Scale (BITS) is the most frequently used instrument to evaluate unnecessary and unreasonable tasks^4,13^. To date, nevertheless, few psychometric property studies have been conducted^13–15^. The contribution of the current study is its simultaneous application of a classical test theory and an item response theory (IRT) to assess the validity of BITS. IRT is a family of associated mathematical models that relate latent traits (e.g., perceived illegitimate tasks) to the probability of responses to items in an assessment. Thus, IRT is used to evaluate the item performance and precision of the scale measuring a latent trait^16^. It is the first study to report item functioning of BITS using IRT consisting of two conceptually separate dimensions capturing unnecessary and unreasonable tasks.

### Illegitimate tasks in the workplace

In line with SOS theory^7,8^, illegitimate tasks decrease employees’ self-esteem^2,11,17,18^, as they may imply a lack of appreciation for their professional role^5,19^. Therefore, illegitimate tasks threaten one’s personal and social self, and professional identity^7,8^. In particular, intrinsically motivated employees suffered more from illegitimate tasks when they were not recognised for their job efforts^14^. Illegitimate tasks contribute to aversive and counterproductive behaviours^4,20^ or poorer job performance^10,21^. Illegitimate tasks can be accompanied by other job demands (e.g., work overload) which threaten both the wellbeing and health of employees^22^. They are also related to a higher level of burnout^23,24^, lower job satisfaction^12,25,26^, work engagement and meaning of work^27^, and higher turnover intentions^28,29^.

Significantly, illegitimate tasks have been identified as a severe problem across various workplaces, including the healthcare sector^6,15,30,31^, higher education^32^, IT professionals^33^, teachers^5^, engineers^17^, administrative staff^11^, blue-collar workers^34^, and also Red Cross volunteers^29^.

In terms of other effects, illegitimate tasks may also be transferred to the domain outside work. Due to this, illegitimate tasks have been shown to be associated with work-family conflict^13,24^, which may be exacerbated by psychological detachment^35^. Moreover, illegitimate tasks are related to biological stress indicators, such as worse sleep quality^36^ and a higher level of cortisol, especially among male employees who evaluated their health as poor^37^.

Illegitimate tasks constitute two distinct facets: unnecessary and unreasonable tasks^2,4^. For example, unnecessary tasks are frequently observed^30^ in the healthcare sector. However, unreasonable tasks, such as tasks outside one’s occupational role, are also widely reported^6^. Unnecessary tasks are related to job dissatisfaction^38^, while unreasonable tasks consume time and energy^30,39^. Furthermore, unnecessary tasks result in a fluctuation of negative affect within individuals, while unreasonable tasks lead to negative affect at the between-person level^18^. Unnecessary tasks predict a higher level of distress, while unreasonable tasks cause a broader spectrum of mental health issues, such as distress, anxiety, and depression^40^.

Concluding, illegitimate tasks are commonplace at work. They may negatively affect the effectiveness of an organisation. They may also harm employee wellbeing and organisational behaviour and even encroach on other areas of employees’ lives.

### Bern Illegitimate tasks scale

Illegitimate tasks are usually measured with BITS, which consists of two conceptually separate dimensions capturing unnecessary and unreasonable tasks. Initially, BITS comprised nine items that were classified into two dimensions^13^. Later, however, their number was reduced to eight^4^. The two dimensions are moderately correlated^2,13,29^.

The popularity of BITS is underlined by the fact that it has been translated into several languages, including English^11^, Swedish^10,41^, Norwegian^39^, Finnish^6,27^, Turkish^42^, Indian^43^, Chinese^21,35^ and Spanish^15^. Existing studies have mostly focused on the scale’s convergent and discriminant validity. Moreover, most studies have shown that BITS has good internal consistency with regard to both subscales (unnecessary and unreasonable tasks)^15^.

However, few studies have demonstrated the psychometric properties of BITS in detail based on classical test theory. Using confirmatory factor analysis (CFA), Jacobshagen^13^, Muntz and Dormann^14^, and Portilla et al.^15^ observed two moderately correlated subscales. Even though some studies on the construct validity of BITS have conceived illegitimate tasks as having only a single construct^26,27^, they concordantly emphasise that this scale captures two substantially different types of illegitimate tasks.

As mentioned above, the original version of BITS consisted of nine items^13^. Some data indicated that Item 4: ‘Do you have work tasks to take care of which keep you wondering if they would not exist (or could be done with less effort), if some other people made less mistakes?’ loaded on both factors^13^ (see Table 9, p. 75). Thus, Jacobshagen suggested that Item 4 should be omitted, and Semmer et al.^4^ started applying the eight-item version of BITS. Unfortunately, it was not emphasised enough that Item 4 had been removed, and a sole description of four items for both unnecessary and unreasonable tasks was revealed. Consequently, this change was confusing to researchers, who have been applying different versions since. The nine-item BITS is still used^25,32^. Moreover, a few reports of factor analysis revealed that occasionally Item 5 was removed^25,44^. In addition, BITS was reduced to a few items^45^ or a single-item scale for unnecessary and unreasonable tasks^23^. Furthermore, we have found different response formats in the existing studies. In addition to the original five-point response format scale, we have also observed a four- and seven-point scale ranging from ‘strongly disagree’ to ‘strongly agree’^17,20,23,42^.

### The current study

In the 16-year history of BITS, there have been too few solid reports on the psychometric properties of the scale. It seems necessary to test if the eight items of BITS sufficiently measure the unidimensionality of the construct or two separate dimensions. Moreover, no published studies have investigated the item and scale functioning of BITS using IRT. Therefore, to date, our study is the first to report the item and scale functioning of this instrument using IRT.

An overarching purpose of the current study was to evaluate the factor structure of BITS, to determine whether the items and dimensions of BITS function well, and to validate the Polish version of BITS as an important job-related stressor.

We have formulated the following research questions:

*RQ1* What is the structure of BITS, i.e., how many factors does it represent and is it homogeneous (unidimensional)?

*RQ2* How effectively does each item discriminate between employees with different levels of illegitimate tasks (i.e., unnecessary tasks and unreasonable tasks), as well as.do the items adequately measure illegitimate tasks across low to high levels?

*RQ3* What is the construct validity, namely, convergent and discriminant validity, of the Polish version of BITS?

### Methods

To assess the psychometric properties of BITS we used two heterogeneous samples of employees. In sample 1, first we examined the factor structure of BITS and then assessed item discrimination and local dependence. In sample 2, we replicated the construct validity and examined the convergent and discriminant validity, as well as the reliability of BITS.

### Participants and Procedure

#### Sample 1

To conduct a two-parameter logistic model (2PLM) IRT test with a graded response model (GRM)^46^, the recommended minimum sample size is 500 to 750 participants for 10–20 items^47^. In 2019, 966 fully completed protocols were received. One outlier by having a high value of the Mahalanobis distance (p < 0.001) was detected. Thus, the sample consisted of 965 employees at Polish organisations in the education, public administration and IT sectors. Respondents (65% women) were aged between 21 and 68 years with a mean age of 42.4 years (*SD* = 10.2 years), and they had job tenures of 1 to 40 years. Most had high levels of education (bachelor’s or master’s degree).

#### Sample 2

In 2020, 821 fully completed protocols were obtained (response rate 68%). 18 multivariate outliers were excluded. Thus, the final sample consisted of 803 employees at different Polish organisations in the healthcare, education, public administration and IT sectors. Participants (59% women) were aged between 23 and 66 years with a mean age of 43 years (*SD* = 10.2 years). They had an average job tenure of 19.7 years (*SD* = 10.8 years, range 2–45) and an average job tenure in their current organisation of 12.9 years (*SD* = 10.1, range 0.5–41).

### Ethics statement

The study was performed in line with the principles of the Declaration of Helsinki. All data were collected using anonymous online surveys. All participants were informed of the nature of the current study and gave their informed consent to participate. According to national recommendations, issued by the Psychological Committee of the Polish Science Academy (21st June 2013) and the National Science Centre, ethics approval to conduct this study was not required. More details about the study procedure are presented in the Supplementary material.

### Measures

We took the original Bern Illegitimate Tasks Scale** (**BITS)^13^ instrument including nine items (all of which are positively worded) in order to replicate previous findings from past research, and we clarified how each item functioned. Unnecessary tasks are assessed on BITS with five items. Each item is introduced with the lead-in question, ‘Do you have work tasks to take care of which keep you wondering if…’, followed by phrases such as ‘…they have to be done at all?’ There are four items assessing unreasonable tasks, and each of these is introduced with the lead-in question, ‘Do you have work tasks to take care of which you believe…’, followed by phrases such as ‘…should be done by someone else?’ Responses are marked on a five-point Likert scale ranging from 1 (never) to 5 (frequently). Higher scores indicate that employees are more often required to do unnecessary and unreasonable tasks.

A six-stage translation and adaptation process was used to adapt BITS from English to Polish^48^. This process included forward translation into Polish by two different translation agencies, synthesis, back translation from Polish to English, harmonisation, cognitive interviews, revisions, and pilot data sampling. The Polish version can be found in the Supplementary material.

To assess the convergent and discriminant validity of BITS (sample 2), we applied related constructs such as job demand (work overload) and outcomes (work performance and occupational wellbeing, i.e., job burnout and job satisfaction). Instruments to evaluate these constructs are reported in Table 1.

**Table 1:** Study instruments: item number, sample item, response format and citation.

Constructs and citation	Item	Sample items	Response format
Work overload^49^	4	Do you have too much work to do?	5-point (1 = never, 5 = always)
Burnout Assessment tool^50^	23	I want to be active at work, but somehow I am unable to manage	5-point (1 = never, 5 = always)
Job satisfaction^51^	1	To what extent are you satisfied with your job?"	5-point (1 = very dissatisfied, 5 = very satisfied)
Work performance^49^	1	How would you rate your current job performance?	11 point (from 0 to 10, the higher, the better)

### Statistical analyses

#### Preliminary analyses

Scores on the total BITS and its two dimensions were calculated according to the manual^13^. Using sample 1, the exploratory factor analysis (EFA; the principal axis factoring extraction method with oblimin rotation) and parallel analysis (PA) identified two factors. Each item belongs to one of the two factors. The factor loadings for the items were above 0.68, with the exception of Item 4 (0.44) (see Supplementary Table S1). Indeed, the content of this item refers to both unnecessary and unreasonable tasks. IRT for the original nine-item BITS was also carried out and, following Baker’s^16^ cut-offs, almost all items on the unnecessary tasks dimension were designated very high, whereas Item 4 was moderate in terms of its precision to discriminate between respondents with different levels of illegitimate tasks. Thus, Item 4 was relatively different. Considering jointly the preliminary analyses, Item 4 was removed from further analyses. Consequently, two subscales containing four items each were assigned to the analyses (unnecessary tasks: Items 1–3, 5; unreasonable tasks: Items 6–9).

#### Classical test theory analyses

To ensure that illegitimate tasks measured by items of BITS met the IRT assumption of unidimensionality, EFA and PA^52^ were conducted. Using EFA, the most commonly used indicators that support the unidimensionality assumption include having at least 20% of the variance on the first factor or having a ratio between the eigenvalues of the first factor compared to the next factor that is greater than 4^53^.

Factor validity was also assessed with CFA based on a maximum likelihood estimation method in sample 1, and we replicated these findings in sample 2. The assumptions of data being continuous with multivariate normal distribution were met. The goodness-of-fit of the CFA model was assessed by the chi-squared test (χ^2^), comparative fit index (CFI), root mean square error of approximation (RMSEA), standardised root mean square residual (SRMR) and Bayesian information criterion (BIC). Values of CFI > 0.90^54^, RMSEA and SRMR < 0.08, and a lower BIC^55^ indicate an acceptable goodness-of-fit. Measurement reliability was established using two coefficients, which were Cronbach’s alpha and composite reliability (CR) with the value > 0.70^56^.

Using sample 2, convergent validity was measured in terms of average variance extracted (AVE) with the value > 0.50 for each latent variable^57^. Discriminant validity was assessed by examining whether AVE was higher than the squared coefficients of the bivariate correlation between work overload, job burnout, job satisfaction and work performance^57^. Construct validity was assessed by running multiple linear regressions (enter method). SPSS and AMOS softwares were used to conduct the statistical analyses.

#### Item response theory analyses

Before applying IRT models with GRM, we evaluated the basic assumption of the model’s unidimensionality, monotonicity, and item local independence. The 2PLM IRT analyses were conducted using IRTPRO. Given BITS’ five response categories, a GRM was selected^46^. A key assumption of the GRM is that scores for each item are ordered consistently across items, such that the lowest response category (labelled ‘never’) is indicative of the lowest level of θ (where θ indicates different levels of the illegitimate tasks, measured by BITS). The GRM estimates a common item discrimination parameter for each item (*a*) and also estimates the location or difficulty/severity threshold parameters (*b*_i_) for each response category within the item^58^. Item discrimination describes how well an item can differentiate between examinees at different trait levels. Each item of BITS has an average discrimination parameter *a* across five response categories. A higher *a* value indicates that an item discriminates more precisely between respondents with different levels of θ. Each item was evaluated in terms of its function relative to the other items, which was assessed by both its scale score and its contribution to the overall information gathered from each scale score^59^. Item difficulty is known as “location” on the difficulty range (the location on the latent trait) and describes where the item functions best along the trait scale, namely, lower values will be expected to be endorsed at lower trait levels. Difficulty threshold parameters *b*_*i*_ are expressed as the standard deviation away from the mean of the value 0, and have a common range of − 3 to + 3^59^. Both discrimination and difficulty threshold parameters have been represented graphically as item characteristic curves (ICCs). In addition, measurement invariance of the scale between men and women was estimated by differential item functioning (DIF). Gender differences of the discrimination and difficulty parameters for items were examined using chi-squared tests.

These two approaches, i.e., classical test theory modelling with CFA and modelling using IRT, reinforce each other. CFA is confirmative and able to test the conceptual model of BITS, while IRT produces sample-independent estimates of parameters.

## Results

### Factor structure of BITS

The total BITS (8-item) failed to sufficiently meet the assumptions of unidimensionality based on the ratio between the eigenvalues of the first factor and the next factor being greater than 4^53^. The results of the PA suggested two factors (see Supplementary Table S2).

The extraction of two factors using EFA showed that none of the items cross-loaded on the second factor with a value greater than 0.30 (see Supplementary Table S3). Therefore, two factors were retained as two conceptually-derived dimensions (four items each), which each showed good reliability (Table 2) and supported unidimensionality. Table 2 also presents descriptive statistics of the Polish version of BITS.

**Table 2:** Descriptive statistics of the Polish version of the BITS.

Composite scale score	No items	Item	*M*/*SD*	Sk	K	α/*M*_iic_	Correlation with Illegitimate tasks	% variance first factor; second factor	First eigenvalue /Second eigenvalue = Ratio	UD	Factor correlation (EFAs)
Illegitimate tasks	8	1–3, 5–9	2.93/0.75	0.01	− 0.12	0.89/0.50	–	57;16	4.53/1.26 = 3.60		0.63
Unnecessary tasks	4	1–3, 5	3.10/0.84	− 0.04	− 0.14	0.87/0.64	0.88	73;13	2.91/0.50 = 5.82	Yes	1-factor
Unreasonable tasks	4	6–9	2.75/0.86	0.18	− 0.24	0.87/0.62	0.89	72;12	2.87/0.50 = 5.74	Yes	1-factor

Sample 1 (*N* = 965). Sk = Skewness. K = Kurtosis. *M*_iic_ = Mean inter-item correlation. UD = Unidimensionality according to Reeve et al.^54^.

We also assessed factor validity for BITS using CFA. We analysed two models: a single-factor model and a model of two correlated latent factors (Table 3). The findings revealed that the modified model with the two correlated latent factors, including two pairs of error correlation (Item 1 and Item 2, as well as Item 8 and Item 9), yielded an excellent fit. Those factors were moderately correlated (0.66) (see Supplementary Fig. S1A). This result confirmed the result from the PA, indicating that the eight-item BITS, applied on the current sample, comprises two separate dimensions, and therefore separate analyses were performed on both IRT models, and validation analyses.

**Table 3:** Confirmatory factor analysis of the single—and two- factor model of BITS.

Model	Χ^2^ (df)	CFI	SRMR	RMSEA (90%CI)	BIC	Δχ^2^	Δdf
Sample 1 N = 965							
M1: single factor model	1117.553 (20)	0.750	0.106	0.239 (0.227, 0.251)	1227.507	–	–
M2: correlated two factor model	179.644 (19)	0.963	0.044	0.094 (0.810, 0.106)	179.644	937.909***	1
M3: modified correlated two factor model	43.404 (17)	0.994	0.021	0.040 (0.025, 0.055)	173.974	136.240***	2
Sample 2 N = 803							
M1: single factor model	1104.742 (20)	0.725	0.112	0.260 (0.247, 0.273)	1211.756	–	–
M2: correlated two factor model	248.706 (19)	0.942	0.066	0.123 (0.109, 0.137)	362.408	849.348***	1
M3: modified correlated two factor model	34.225 (17)	0.996	0.019	0.036 (0.018, 0.053)	161.304	201.104***	2

CFI = confirmatory fit index, SRMR = standardized root mean square residual, RMSEA = root mean square error of approximation, 90%CI = 90% confidence interval, BIC = Bayesian information criterion.

*** *p* < 0.001.

### Item functioning

Table 4 shows that discrimination parameter *a* ranged from 1.96 to 4.93 for items relating to unnecessary tasks and from 2.42 to 3.27 for those relating to unreasonable tasks. Following Baker’s^16^ cut-offs, all items were designated ‘very high’ (≥ 1.69)*.* Therefore, the items were deemed able to adequately distinguish between employees with different levels of unnecessary and unreasonable tasks.

**Table 4:** Item Response Analysis of the Polish version of the BITS.

Item	*a*/*s.e*	Quality of *a*	*b*_1_	*b*_*2*_	*b*_3_	*b*_4_	p for DIF*	
							*a*_*i*_	*b*_*i*_
Unnecessary tasks								
Do you have work tasks to take care of which keep you wondering if …								
2…they make sense at all?	4.93/0.41	Very high	− 1.75	− 0.59	0.56	1.46	0.34	0.44
1…they have to be done at all?	4.70/0.38	Very high	− 1.84	− 0.69	0.59	1.61	0.19	0.29
3…they would not exist (or could be done with less effort), if it were organized differently?	2.09/0.12	Very high	− 2.21	− 0.89	0.58	1.91	0.04	0.28
5…they just exist because some people simply demand it this way?	1.96/0.11	Very high	− 2.18	− 0.83	0.34	1.59	0.34	0.12
Unreasonable tasks								
Do you have work tasks to take care of, which you believe …								
7…are going too far, which should not be expected from you?	3.27/0.23	Very high	− 1.47	− 0.34	0.73	1.75	0.04	0.11
9…are unfair that you have to deal with them?	2.98/0.20	Very high	− 1.04	0.05	1.08	2.15	0.39	0.21
6…should be done by someone else?	2.51/0.15	Very high	− 2.16	− 0.79	0.47	1.75	0.44	0.38
8…put you into an awkward position?	2.42/0.15	Very high	− 1.21	0.01	1.25	2.20	0.67	0.79

Sample 1 (*N* = 965). *a* = discrimination parameter. Items were ranked by the *a* value. Very high ≥ 1.69. *b*_1_-*b*_4_ = item difficulty/threshold parameters. Shadowed *b*_*i*_ values > 95 percentiles. DIF = differential item functioning. * *p* values of chi-square tests to examine DIF with respect to gender-specific discrimination and difficulty parameters (Bonferroni adjusted for multiple comparisons 0.05/4 = 0.0125 indicating that no significant DIF were found).

**Figure 1:**
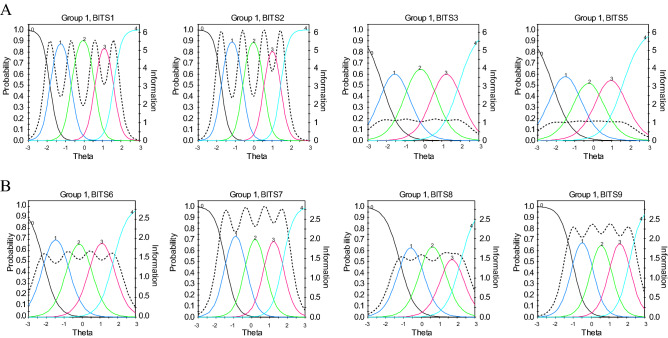
Item characteristics curves (ICCs, colored lines), combined with item information functions (IIFs, dashed lines) for the unnecessary tasks’ subscale (**A**) and for the unreasonable tasks’ subscale (**B**) of the Polish version of the BITS (Sample 1 N = 965).

An inspection of the item difficulty parameters *b* indicates that both dimensions of illegitimate tasks in the Polish version of BITS adequately measured these traits across low to high levels. DIF showed that all items had measurement invariance between males and females. ICCs combined with IIFs for the four unnecessary task items and four unreasonable task items in the Polish version of BITS are presented in Fig. 1.

**Figure 2:**
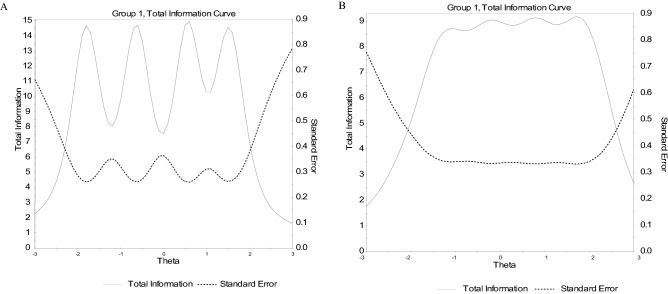
Test information function (TIF) of the dimension of unnecessary tasks (**A**) and the unreasonable tasks (**B**) of the Polish version of the BITS under the graded response model (GRM) (Sample 1 N = 965). Note. Latent trait θ is shown on the horizontal axis, and the amount of information and the standard error yielded by the test at any trait level are shown on the vertical axis. Ranging from about − 2.30 *SD*s below the mean to about 2.00 *SD*s above the mean, the amount of test information was at least 6.5 (which yields a standard error of estimate about 0.38). We can interpret the information magnitude by computing the associated reliability (*r* = 1–1/information). Thus, reliability was equal to or greater than 0.85, and within the range described. Note. Latent trait θ is shown on the horizontal axis, and the amount of information and the standard error yielded by the test at any trait level are shown on the vertical axis. Ranging from about -2.00 *SD*s below the mean to about 2.50 *SD*s above the mean, the amount of test information was at least 4.8 (which yields a standard error of estimate about 0.47). We can interpret the information magnitude by computing the associated reliability (*r* = 1–1/information). Thus, reliability was equal to or greater than 0.73 within the range described.

Concerning specific reliability, the test information function indicated that both factors were sufficiently informative (i.e., reliable) for a broad range of the trait illegitimate tasks (see Fig. 2).

### Further validity of BITS: replication in sample 2

We replicated construct validity for BITS using CFA in sample 2 (Table 3). The single-factor model showed a poor fit to the data and was worse than the two-factor models. A modified model with the two correlated latent factors obtained an excellent fit. Those factors were moderately correlated (0.67) (see Supplementary Fig. S1B).

Convergent validity was assessed with AVE, and the value for both factors in the model exceeded the criterion of 0.50 (0.62 for unnecessary and unreasonable tasks). In sample 2 unnecessary tasks were more frequently observed than unreasonable tasks (*t(802)* = 11.03, *p* < 0.001).

Discriminant validity was assessed with the Fornell–Larcker^57^ criterion in regard to work overload, occupational wellbeing (i.e., job burnout and job satisfaction) and work performance. Descriptive statistics, reliabilities, and correlations for study variables are presented in Table 5.

**Table 5:** Descriptive statistics, reliabilities, and correlations for study variables.

Variables	*M*	*SD*	*Sk*	*K*	1	2	3	4	5	6
1. Unnecessary tasks	2.97	0.78	− 0.04	0.02	(0.89)					
2. Unreasonable tasks	2.68	0.82	0.23	−0 .07	0.57***	(0.87)				
3. Work overload	3.59	0.62	− 0.37	0.02	0.34***	0.46***	(0.75)			
4. Job burnout	2.31	0.59	0.32	− 0.01	0.44***	0.44***	0.40***	(0.95)		
5. Job satisfaction	3.70	0.75	−0 .90	1.76	− 0.33***	− 0.32***	− 0.21***	− 0.47	−	
6. Work performance	7.75	1.50	− 1.12	2.40	− 0.21***	− 0.11**	− 0.06	− 0.39***	0.32***	−

Sample 2 *N* = 803. *Sk* = Skewness. *K* = Kurtosis. Scale reliabilities listed along diagonal.

*p < 0.05, **p <0 .01, ***p < 0.001.

AVE for unnecessary tasks (0.62) was higher than the value of its squared correlations with work overload (0.12), job burnout (0.19), job satisfaction (0.11) and work performance (0.04). In addition, AVE for unreasonable tasks (0.62) was higher than the value of its squared correlations with work overload (0.21), job burnout (0.11), job satisfaction (0.10) and work performance (0.01). Therefore, both types of illegitimate tasks can be regarded as distinct from measures assessing the level of work overload, occupational wellbeing and work performance. Further to this, we evaluated the reliability of BITS. The Cronbach’s alpha value was 0.89 for unnecessary tasks and 0.87 for unreasonable tasks, and similarly, the CR was 0.87 for both subscales. Thus, it confirmed the internal consistency and reliability of BITS.

Construct validity was also assessed by running multiple linear regressions, including work overload and both types of illegitimate tasks as predictors, and two indicators of occupational wellbeing (namely, job burnout and job satisfaction), as well as work performance (Table 6).

**Table 6:** Job demands, occupational well-being and work performance—results of multiple linear regressions.

Job demands	Job burnout				Job satisfaction				Work performance			
	*B*	*SE*	*β*	*t*	*B*	*SE*	*β*	*t*	*B*	*SE*	*β*	*t*
Constant	0.59	0.11		5.35***	5.00	0.15		32.69***	8.85	0.32		27.47***
Unnecessary tasks	0.20	0.03	0.26	7.15***	− 0.21	0.04	− 0.22	− 5.36***	− 0.42	0.08	− 0.22	− 5.14***
Unreasonable tasks	0.13	0.03	0.18	4.66***	− 0.15	0.04	−0 .17	− 3.90***	0.03	0.08	0.02	0.35
Work overload	0.22	0.03	0.23	6.70***	− 0.08	0.05	−0 .06	− 1.70	0.02	0.10	0.01	0.18
*R*^*2*^	0.29				0.14				0.04			
*F(df)*	106.43*** (3, 799)				42.87 (3, 799)***				11.94*** (3, 799)			

Sample 2* N* = 803. *B* = Unstandardized Regression Coefficients. *β* = standardized Regression Coefficients.

*p < 0.05, **p < 0.01, ***p < 0 .001.

Unnecessary tasks, unreasonable tasks, and work overload explained 29% of the variance in job burnout. Additionally, both unnecessary and unreasonable tasks, but not work overload, explained 14% of the variance in job satisfaction. Therefore, as expected, both types of illegitimate tasks explain more variance in job burnout, which is a negative indicator of wellbeing, compared with job satisfaction, which is a positive indicator of wellbeing. Moreover, unnecessary tasks, but not unreasonable tasks or work overload, explained 4% of the variance in work performance.

## Discussion

Combining the classical test and item response theories, our results showed that BITS is *not* a unidimensional scale. It measures two conceptually different separate dimensions, comprising four items each. IRT analyses showed that all items on each factor on different levels were very highly informative (reliable). All levels of the unnecessary and unreasonable tasks were reliably captured by the BITS items, suggesting that the questionnaire has very good psychometric properties. Moreover, BITS had measurement invariance with respect to gender-specific discrimination and difficulty parameters. Additionally, the results from validity tests revealed that both dimensions of unnecessary and unreasonable tasks were distinct from other work overload and occupational wellbeing indicators.

The present study investigated for the first time the psychometric properties of an eight-item BITS, using IRT, to provide evidence that this questionnaire reliably measures illegitimate tasks among employees without producing biased data. In addition, a new language version was verified. Thus far, a German and a Spanish version of BITS have been verified using CFA^13–15^. Therefore, our study links advantages of the classical test and item response theories.

We used IRT to analyse the extracted two factors rather than the single scale of illegitimate tasks, as the latter did not meet the unidimensional criterion. Moreover, the results of parallel analysis and CFA, as well as the findings of past research on BITS^2,4,13–15^, showed that the construct of illegitimate tasks consists of two dimensions, namely, unnecessary and unreasonable tasks.

This study also provides evidence that Item 4 may be excluded from the original version of BITS. Indeed, using classical test theory, this item obtained weak validity, psychometrically and semantically. Conceptually, it refers to both subscales (i.e., unnecessary and unreasonable tasks). IRT analysis has shown Item 4 had moderate discrimination power^16^, and due to this, this item produced relatively lower and flatter information curves. Additionally, scarce previous research on BITS has resulted in researchers applying the eight-item BITS, although Item 4 was not always excluded^25,44^. Therefore, different content versions are implemented. Finally, some studies have been based on different response formats^17,20,23,42^. Due to this confusion in the literature, the existing findings cannot be accurately compared. Overall, we present new evidence for improving BITS, which replicates in great depth previous findings^4,13^.

There are no previous IRT findings on BITS to compare with the current study’s results. However, significantly, all eight items with very high discrimination power indicate that the items of the Polish version of BITS can adequately distinguish between people with different levels of illegitimate tasks. Moreover, in our study, Item 2 and Item 1 (for unnecessary tasks) and Item 7 (for unreasonable tasks), demonstrated the highest discrimination power (see Table 4 and also observe the dashed lines in Fig. 1). Thus, we may propose that in future studies requiring single items, e.g., in diary studies, researchers may use the three mentioned items (1, 2 and 7). However, we have found a diary study^23^ that used Item 3 and Item 6 as representative of the unnecessary and unreasonable tasks, respectively. No rationale was presented for using these items. We also suggest that a reduced scale, with only three items, can be useful in screening tests or surveys comprising a multitude of scales. However, fully established evidence requires further IRT analysis among other language versions.

Convergent and discriminant analyses revealed that the Polish version of BITS is valid for both its factors. Furthermore, BITS is a reliable instrument, as earlier studies have found^14,15,24^. Our study adds to prior research by presenting reliability on the whole scale range, using IRT (see Figs. 1 and 2). The results of our study, like previous studies, confirmed that unnecessary and unreasonable tasks are separate constructs in terms of stressful job demands. Similar to existing studies^12,24,26^, illegitimate tasks were moderately correlated with work overload and jointly predicted occupational wellbeing. However, both dimensions of illegitimate tasks in our sample contributed more to occupational wellbeing beyond and above work overload. What is more important, illegitimate tasks related to job burnout to a greater extent than job satisfaction. It is consistent with the assumptions of Job Demands-Resources theory^60^, that excessive social demands negatively affect people’s health more than decrease their motivation.

Additionally, we have observed that unnecessary tasks occurred more often than unreasonable tasks among Polish employees. The same was noted for Swedish employees^30^. Moreover, our findings demonstrated that only the unnecessary tasks were negatively related to employees’ self-rated work performance. However, to a lesser extent than they affected their wellbeing. Unnecessary tasks go hand-in-hand with ‘red tape’, are more often assigned at random^17^ and may create unproductive organisational behaviour and diminish the meaningfulness of work among teachers^5^, administrative staff^11^ and IT specialists^33^.

Our study has some shortcomings. The samples were not representative of the employed Polish population, and they were limited to four economic sectors, i.e., IT, administration, education and healthcare. Nonetheless, samples included respondents from each of Poland’s 16 voivodeships. We should emphasise that less-educated employees were underrepresented. In addition, self-reporting methods were used, therefore in further research it is advisable to obtain data from a variety of sources, as was done by Muntz and Dorman^14^. Above all, the cross-sectional design is an important limitation for estimating the predictor-outcomes relationship and, also, for controlling the common method variance^59^. However, Harman’s single-factor method showed that CMV was not a problem in this study (the first factor explained 35.1% of the total variance in sample 2, which constituted less than half of the covariance among measures).

Our findings may have practical implications. BITS has proven to be a valid and reliable scale that can be used in managerial practice. Illegitimate tasks are widespread, therefore this is still a real problem in work management^5,6,17^. Thus, a valuable diagnostic instrument to identify illegitimate tasks is expected. Our findings provided new evidence that BITS is an exceptional scale. In addition, based on the IRT results, we may suggest that a few items of BITS could be useful during screening examination and diary surveys to control the rationale of work tasks. And above all, in day-to-day interviews (e.g., during staff interviews), to help managers focus on a qualitative understanding of illegitimate tasks.

We conclude that the Polish version of BITS, completed by employees from different Polish organisations, is best measured by its two dimensions, namely, unnecessary and unreasonable tasks, which include four items each. The validity and reliability results of BITS demonstrate that the scale is outstanding and worth applying in scientific research and managerial practice.

## Electronic supplementary material

Below is the link to the electronic supplementary material.

Supplementary Information.

Supplementary Information.

## Data Availability

The data underlying the results presented in the study are available on Mendeley (https://doi.org/10.17632/7wfgz62xgs).

## References

[1] Baran BE, Woznyj HM (2020). Managing VUCA: The human dynamics of agility. Organ. Dyn..

[2] Semmer NK (2015). Illegitimate tasks as a source of work stress. Work Stress.

[3] Ding H, Kuvaas B (2022). Illegitimate tasks: A systematic literature review and agenda for future research. Work Stress.

[4] Semmer NK, Tschan F, Meier LL, Facchin S, Jacobshagen N (2010). Illegitimate tasks and counterproductive work behavior. Appl. Psychol. Int. Rev..

[5] Faupel S, Otto K, Krug H, Kottwitz MU (2016). Stress at school? A qualitative study on illegitimate tasks during teacher training. Front. Psychol..

[6] Kilponen K, Huhtala M, Kinnunen U, Mauno S, Feldt T (2021). Illegitimate tasks in health care: Illegitimate task types and associations with occupational well-being. J. Clin. Nurs..

[7] Semmer NK, Jacobshagen N, Meier LL, Elfering A, Houdmont J, McIntyre S (2007). Occupational stress research: The “stress-as-offense-to-self“ perspective. Occupational health psychology: European perspectives on research, education and practice 43–60.

[8] Semmer NK (2019). Stress as offense to self: A promising approach comes of age. Occup. Health Sci..

[9] Fila MJ, Eatough E (2020). Extending the boundaries of illegitimate tasks: The role of resources. Psychol. Rep..

[10] Björk L, Bejerot E, Jacobshagen N, Härenstam A (2013). I shouldn’t have to do this: illegitimate tasks as a stressor in relation to organizational control and resource deficits. Work Stress.

[11] Eatough EM, Meier LL, Igic I, Elfering A, Spector PE, Semmer NK (2016). You want me to do what? Two daily diary studies of illegitimate tasks and employee well-being. J. Organ. Behav..

[12] Omansky R, Eatough EM, Fila MJ (2016). Illegitimate tasks as an impediment to job satisfaction and intrinsic motivation: Moderated mediation effects of gender and effort-reward imbalance. Front. Psychol..

[13] Jacobshagen N (2006). Illegitimate tasks, illegitimate stressors: Testing a new stressor-strain concept.

[14] Muntz J, Dormann C (2020). Moderating effects of appreciation on relationships between illegitimate tasks and intrinsic motivation: A two-wave shortitudinal study. Eur. J. Work Organ. Psy..

[15] Portilla DLV, Rosero AG, Alvarado-Villa G, Moncayo-Rizzo J (2021). Psychometric properties of the Bern illegitimate tasks scale-Spanish version. Front. Psychol..

[16] Baker FB (2001). The basics of item response theory.

[17] Pindek S, Demircioğlu E, Howard DJ, Eatough EM, Spector PE (2019). Illegitimate tasks are not created equal: Examining the effects of attributions on unreasonable and unnecessary tasks. Work Stress.

[18] Sonnentag S, Lischetzke T (2018). Illegitimate tasks reach into afterwork hours: A multilevel study. J. Occup. Health Psych..

[19] Pfister IB (2020). Appreciation and illegitimate tasks as predictors of affective well-being: Disentangling within-and between-person effects. J. Work Organ. Psychol..

[20] Zhao L, Lam LW, Zhu JN, Zhao S (2021). Doing it purposely? Mediation of moral disengagement in the relationship between illegitimate tasks and counterproductive work behavior. J. Bus. Ethics.

[21] Ma J, Peng Y (2019). The performance costs of illegitimate tasks: The role of job identity and flexible role orientation. J. Vocat. Behav..

[22] Hirschle ALT, Gondim SMG (2020). Stress and well-being at work: A literature review. Cien Saude Coletiva.

[23] Bianchi R, Manzano-García G, Rolland JP (2021). Is burnout primarily linked to work-situated factors? A relative weight analytic study. Front. Psychol..

[24] Meier LL, Semmer NK (2018). Illegitimate tasks as assessed by incumbents and supervisors: Converging only modestly but predicting strain as assessed by incumbents, supervisors, and partners. Eur. J. Work Organ. Psy..

[25] Ilyas A, Hassan R, Khan A, Khan W (2021). Illegitimate tasks and job satisfaction among employees of micro informal enterprises. Manag. Sci. Lett..

[26] Kottwitz MU, Pfister IB, Elfering A, Schummer SE, Igic I, Otto K (2019). SOS-Appreciation overboard! Illegitimacy and psychologists’ job satisfaction. Ind. Health.

[27] Mäkikangas A, Minkkinen J, Muotka J, Mauno S (2021). Illegitimate tasks, job crafting and their longitudinal relationships with meaning of work. Int. J. Hum. Resou. Man..

[28] Ping ZL, Fu HY, Ye ZX, Zhao S (2021). Illegitimate tasks and employees’ turnover intention: A serial mediation model. Front. Psychol..

[29] van Schie, S., Güntert, S. T. & Wehner, T. (2014) How dare to demand this from volunteers! The impact of illegitimate tasks. *Voluntas: International Journal of Voluntary and Nonprofit Organizations* **25**, 851–868, 10.1007/s11266-013-9375-4

[30] Anskär E, Lindberg M, Falk M, Andersson A (2019). Legitimacy of work tasks, psychosocial work environment, and time utilization among primary care staff in Sweden. Scand. J. Prim. Health..

[31] Stein M, Vincent-Höper S, Schümann M, Gregersen S (2020). Beyond mistreatment at the relationship level: Abusive supervision and illegitimate tasks. Int. J. Environ. Res. Public Health.

[32] Bramlage JK, Julmi C, Pereira JM, Jackenkroll B (2021). When enough is enough: modelling the path from unreasonable tasks to the intention to leave academia. Eur. J. High. Educ..

[33] Apostel E, Syrek C, Antoni CH (2018). Turnover intention as a response to illegitimate tasks: The moderating role of appreciative leadership. Int. J. Stress Manage..

[34] Mauno S, Minkkinen J, Shimazu A (2022). Do unnecessary tasks impair performance because they harm living a calling? Testing a mediation in a three-wave study. J. Career Assess..

[35] Zhou ZE, Eatough EM, Che XX (2020). Effect of illegitimate tasks on work-to-family conflict through psychological detachment: Passive leadership as a moderator. J. Vocat. Behav..

[36] Pereira D, Semmer NK, Elfering A (2014). Illegitimate tasks and sleep quality: an ambulatory study. Stress Health.

[37] Kottwitz MU, Meier LL, Jacobshagen N, Kälin W, Elfering A, Hennig J, Semmer NK (2013). Illegitimate tasks associated with higher cortisol levels among male employees when subjective health is relatively low: an intra-individual analysis. Scand. J. Work Env. Hea..

[38] Muntz J, Dormann C, Kronenwett M (2019). Supervisors’ relational transparency moderates effects among employees’ illegitimate tasks and job dissatisfaction: A four-wave panel study. Eur. J. Work Organ. Psy..

[39] Thun S, Halsteinli V, Løvseth L (2018). A study of unreasonable illegitimate tasks, administrative tasks, and sickness presenteeism amongst Norwegian physicians: an everyday struggle?. BMC Health Serv. Res..

[40] Graf-Vlachy L, Sun S, Zhang SX (2020). Predictors of managers’ mental health during the COVID-19 pandemic. Eur. J. Psychotraumato..

[41] Aronsson G, Bejerot E, Härenstam A (2012). Onödiga och oskäliga arbetsuppgifter bland läkare: Samband mellan illegitima arbetsuppgifter och stress kartlagt i enkätstudie. Lakartidningen.

[42] Akyurek, S. S., Can, O. Illegitimate tasks and occupational outcomes: The impact of vertical collectivism. In *Evidence-based HRM: A global forum for empirical scholarship*10.1108/EBHRM-02-2021-0025 (2021).

[43] Ahmed SF, Eatough EM, Ford MT (2018). Relationships between illegitimate tasks and change in work-family outcomes via interactional justice and negative emotions. J. Vocat. Behav..

[44] Aronsson G, Mellner C (2016). Illegitima arbetsuppgifter och identitet-En introduktion. Arbetsmarknad & Arbetsliv..

[45] von Thiele Schwarz U (2021). The work of having a chronic condition: development and psychometric evaluation of the distribution of co-care activities (DoCCA) scale. BMC Health Serv. Res..

[46] Samejima F (1969). Estimation of latent ability using a response pattern of graded scores. Psychom. Monogr. Suppl..

[47] Şahin A, Anıl D (2017). The effects of test length and sample size on item parameters in item response theory. Edu. Sci-Theor Pract..

[48] Brislin RW (1970). Back-translation for cross-cultural research. J. Cross Cult. Psychol..

[49] Schaufeli WB (2017). Applying the job demands-resources model. Organ. Dyn..

[50] Schaufeli WB, Desart S, De Witte H (2020). Burnout assessment tool (BAT)—development, validity, and reliability. Int. J. Environ. Res. Public Health.

[51] Wanous JP, Hudy MJ (2001). Single-item reliability: A replication and extension. Organ. Res. Methods.

[52] O’Connor BP (2020). SPSS and SAS programs for determining the number of components using parallel analysis and Velicer’s MAP test. Behav. Res. Meth. Ins. C..

[53] Reeve BB (2007). Psychometric evaluation and calibration of health-related quality of life item banks: Plans for the patient-reported outcomes measurement information system (PROMIS). Med. Care.

[54] Hu LT, Bentler PM (1999). Cutoff criteria for fit indexes in covariance structure analysis: Conventional criteria versus new alternatives. Struc. Equ. Model..

[55] Kline RB (2015). Principles and practice of structural equation modeling.

[56] Hair JF, Hult GTM, Ringle C, Sarstedt M (2014). A primer on partial least squares structural equation modeling (PLS-SEM).

[57] Fornell C, Larcker DF (1981). Evaluating structural equation models with unobservable variables and measurement error. J. Mark. Res..

[58] Penfield RD (2014). An NCME instructional module on polytomous item response theory models. Educ. Meas. Issues Pra..

[59] Podsakoff PM, MacKenzie SB, Lee J-Y, Podsakoff NP (2003). Common method biases in behavioral research: a critical review of the literature and recommended remedies. J. Appl. Psychol..

[60] Bakker AB, Demerouti E (2017). Job demands–resources theory: taking stock and looking forward. J. Occup. Health Psych..

