# Supplementary material for: Psychometric properties of the Bern illegitimate tasks scale using classical test and item response theories
Source: Sci Rep. 2023 May 3;13:7211. doi: 10.1038/s41598-023-34006-0 (PMC10156715; doi:10.1038/s41598-023-34006-0)
Supplement: Supplementary file 1 — Supplementary Information. [file 41598_2023_34006_MOESM1_ESM.pdf]

Supplementary material to Psychometric Properties of the Bern Illegitimate Tasks Scale Using Classical Test and Item Response Theories

Beata Aleksandra Basinska and Anna Maria Dåderman

Samples and study procedure

First phase. This study is part of the large research project 'Job burnout: new conceptualisation in a longitudinal multilevel approach' the aim of which is to extend the theory of burnout syndrome, including its multi-faceted nature. To recruit respondents and conduct the online survey, the professional research company ASM was engaged. Firstly, ASM sent invitations to organisations operating in four branch sectors (education, teaching, IT and healthcare) in each of Poland's 16 voivodeships. The mentioned sectors were consistent with the primary aim of the large project. With digital expansion, traditional risk factors for burnout (emotional demands and workload) may be accompanied by cognitive overload and increased illegitimate tasks. Thus, new job demands could be important for professionals and could result in a growth in job burnout.

Next, people interested in participating contacted ASM directly. They were screened regarding three conditions: being employed full-time, for over one year, and in one of the four branch sectors. A pilot study was carried out among 30 employees.

The study was performed in line with the principles of the Declaration of Helsinki. All data were collected using anonymous online surveys. All participants were informed of the nature of the current study and gave their informed consent to participate. Participation was completely voluntary, and respondents could withdraw at any time. Finally, fully completed protocols (without missing data) were taken into account.

Second phase. The first online survey (CAWI) was conducted between March and May 2019. Among 966 fully completed protocols, we detected one outlier by having a high value of the Mahalanobis distance ($p < .001$). Thus, sample 1 consisted of N = 965 employees employed in the education, public administration and IT sectors.

Third phase. In March 2020, ASM resent invitations to 1201 employees (working in the education, public administration, healthcare and IT sectors). The second online survey was conducted between April 10th and May 24th, 2020. 821 fully completed protocols were

received that fulfilled the inclusion criteria (response rate 68%). In addition, we detected 18 multivariate outliers by having high values of the Mahalanobis distance ($p < .001$). Thus, sample 2 consisted of 803 respondents. The participants were offered a small payment for completing the survey.

Preliminary results of the BITS-9

Table S1. Preliminary analysis of the BITS-9: Factor loadings of exploratory factor analysis

Items	Factor 1	Factor 2
BITS2	.90	-.07
BITS1	.89	-.07
BITS3	.76	.03
BITS5	.68	.07
BITS4	.44	.20
BITS7	-.04	.85
BITS9	-.04	.84
BITS8	.03	.74
BITS6	.12	.69

Results of the Polish version of the BITS (8 items)

Table S2. Actual and random eigenvalues for the Polish version of the BITS

Factor	Actual eigenvalue	Average random eigenvalue	95th percentile random eigenvalue
1	4.529	1.1357347	1.179764
2	1.264	1.0865279	1.118001
3	0.526	1.0484048 [a]	1.074224 [b]
4	0.474	1.0143579	1.037324

Note. Sample 1 (N = 965). 10,000 random datasets were generated based on the same number of items (8) and cases (965) as in the real dataset used for our factor analyses. [a] This value is higher than the actual eigenvalue, indicating that two factors could be retained, according to the rule of ‘average eigenvalue’. [b] This value is higher than the actual eigenvalue, indicating that two factors could be retained, according to the rule of ‘95th percentile eigenvalue’ (see Hayton et al., 2004).

Table S3. Factor loadings of exploratory factor analysis of the Polish version of the BITS

Items	Factor 1	Factor 2
BITS2	.92	-.06
BITS1	.91	-.06
BITS3	.68	.08
BITS5	.65	.10
BITS7	-.03	.85
BITS9	-.04	.84
BITS8	.02	.74
BITS6	.11	.69

Figure S1. Confirmatory factor analysis: two-factor model fit in Sample 1 (A: N = 965) and Sample 2 (B: N = 803).

A

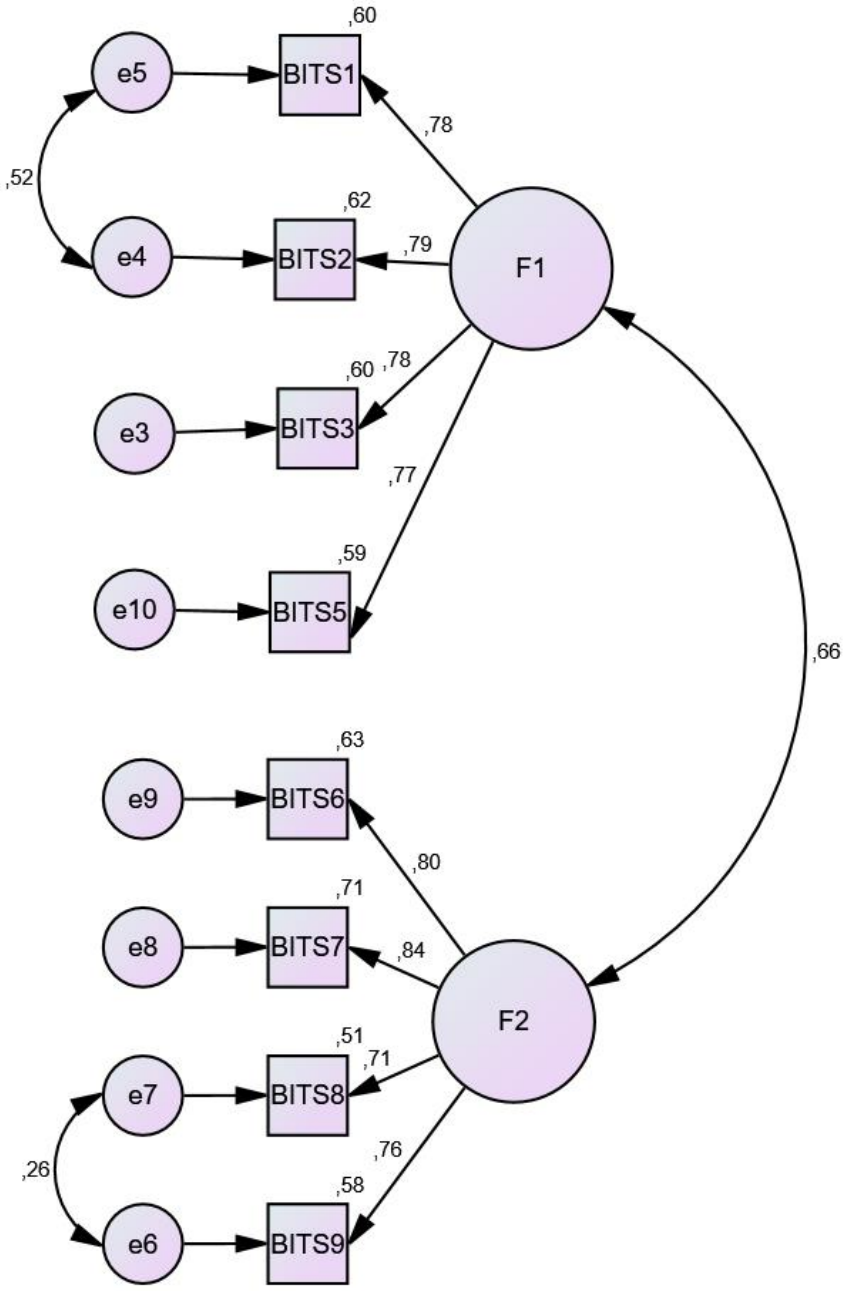

B

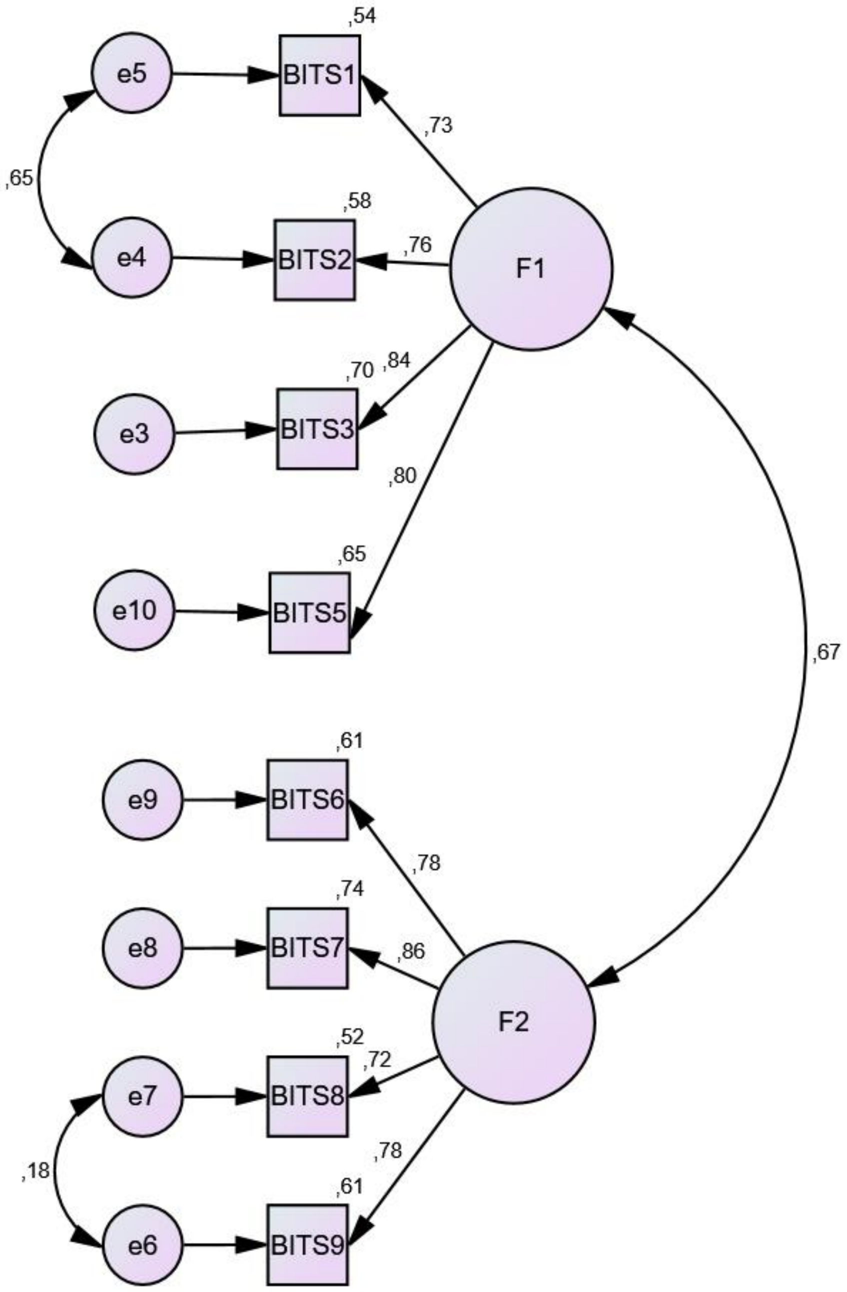

Note. F1 = Unnecessary tasks; F2 = Unreasonable tasks.
