# Supplementary material for: Psychometric properties of the Bern illegitimate tasks scale using classical test and item response theories
Source: Sci Rep. 2023 May 3;13:7211. doi: 10.1038/s41598-023-34006-0 (PMC10156715; doi:10.1038/s41598-023-34006-0)
Supplement: Supplementary file 2 — Supplementary Information. [file 41598_2023_34006_MOESM2_ESM.pdf]

Appendix

The Polish version of Bern Illegitimate Tasks Scale

Polska wersja Berneńskiej Skali Nieuzasadnionych Zadań

Czy masz takie zadania w pracy, którymi się zajmujesz, a zastanawiasz się czy …

	Nigdy	Rzadko	Czasami	Często	Zawsze
1. W ogóle powinny być realizowane?	1	2	3	4	5
2. W ogóle mają sens?	1	2	3	4	5
3. Nie istniałyby (lub były wykonywanie z mniejszym wysiłkiem), gdyby były inaczej zorganizowane?	1	2	3	4	5
5. Istnieją tylko dlatego, że niektórzy wymagają, by były zrobione właśnie w ten sposób?	1	2	3	4	5

Czy masz takie zadania w pracy, którymi się zajmujesz, a uważasz że …

	Nigdy	Rzadko	Czasami	Często	Zawsze
6. Powinny być wykonane przez kogoś innego?	1	2	3	4	5
7. Wykraczają poza twoje kompetencje i nie powinno się ich od ciebie wymagać?	1	2	3	4	5
8. Stawiają Cię w niezręcznej sytuacji?	1	2	3	4	5
9. To niesprawiedliwe, że właśnie Ty musisz je wykonać?	1	2	3	4	5
